# Conceptual art made simple for neuroaesthetics

**DOI:** 10.3389/fnhum.2015.00267

**Published:** 2015-05-12

**Authors:** Alexander Kranjec

**Affiliations:** ^1^Psychology Department, Duquesne UniversityPittsburgh, PA, USA; ^2^Center for the Neural Basis of Cognition, Carnegie Mellon UniversityPittsburgh, PA, USA

**Keywords:** neuroaesthetics, concepts, semantics, Conceptual art, fMRI

Cognitive neuroscience and conceptual art share some interesting common ground. Both are concerned with very basic questions about meaning and objecthood. By focusing on reductionist and contrastive aspects of conceptual art, perhaps neuroscience methods can be applied to the investigation of such questions using everyday objects.

## Making thought visible

*Cognitive neuroscience* seeks to understand basic categories of human behavior, thought, and experience through summary descriptions of neural activity. Like other fields that use empirical methods for investigating the mind, these descriptions can ultimately take verbal or mathematical form. However, explanations in cognitive neuroscience are made using abstract visualizations with a frequency and diversity that surpasses other fields aimed at similar questions. This owes much to the central role that brain imaging methods play in cognitive neuroscience. While fields like psychology and philosophy may be equally engaged in “thinking about thinking,” cognitive neuroscience explicitly seeks to ground the mind in the brain. This is one way of understanding why cognitive neuroscience pushes visualization techniques forward in a way that other empirical investigations of the mind do not. Cognitive neuroscience's main purpose is to understand thought physically using methods that explain it visually.

Although notoriously difficult to define, *conceptual art* (understood here as a *kind* of art with some identifiable characteristics, rather than a particular work of art connected to a formal historical movement) might seem like the inverse of cognitive neuroscience with respect to its relationship with the physical world. Whereas “art” is by default understood as material in kind, conceptual art is often characterized as an art of ideas. Conceptual art is difficult to pin down. Goldie and Schellekens (2013) provide a set of criteria to distinguish conceptual art from more traditional art forms. Generally, they understand conceptual art to be self-reflective and ironic, *against* medium and beauty, and relatively “dematerialized.” That is, conceptual art frequently interrogates traditional ideas of what art is by playfully challenging standards of beauty and medium to the point that much of what constitutes a work of conceptual art is the idea itself. While it should come as no surprise that “ideas” play a critical role in a movement called conceptual art, the characterization of such art as fundamentally dematerialized may be misleading. At least, it might distract from that which cognitive neuroscience and conceptual art have in common.

Both cognitive neuroscience and conceptual art are “bridging disciplines.” Where cognitive neuroscience bridges theory and data from cognitive psychology with an (often abstract, visualized) account of neurophysiology and the brain, conceptual art bridges art theory and practice with an (often abstract, visualized) work of novel art. From this perspective, conceptual art is less a dematerialized form of art, and more a materialized form of art theory. Conceptual artists' work often addresses broad psychological and philosophical questions. A quick survey of works in conceptual art mirrors the organization of topics in a typical introductory cognitive neuroscience textbook. Basic concepts and abstract domains (like language, space, and objecthood) and relatively concrete visual categories of human experience (like words, faces, and colors) take center stage. This shared subject matter reflects deeper, shared ontological and epistemological concerns (e.g., “What is an object?” and “What is a representation?”). Both fields are interested in how concepts are organized psychologically, and by referencing distinct intellectual histories, conceptual art and cognitive neuroscience each engage in describing, abstracting, and visualizing facts about basic categories of mind and experience. Cognitive neuroscience and conceptual art make thought visible.

This is not to say that cognitive neuroscientists are conceptual artists^1^ or *vice versa*. Only that, considering their shared common ground, and what may be a deeper correspondence between them, cognitive neuroscience in general, and the field of neuroaesthetics in particular, could benefit from a greater appreciation of conceptual art. An examination of conceptual art may provide an entry point for investigating the study of meaning in art.

## Meaning in neuroscience

While aesthetics broadly concerns how we produce, perceive, and think about art, neuroaesthetics more specifically investigates the brain's role in such processes (Chatterjee, 2011). A recent review (Chatterjee and Vartanian, 2014) organizes experiments in neuroaesthetics with respect to three general systems of neural circuitry: (1) sensory–motor (2) emotion–valuation, and (3) meaning–knowledge systems. Experiments in neuroaesthetics tend to focus on the first two components of this triad (Vartanian and Goel, 2004; Nadal et al., 2008; Cela-Conde et al., 2011). Neuroimaging (in particular fMRI) studies typically engage participants in tasks that require making preference or “liking” judgments across visual artworks (e.g., paintings) or natural objects (e.g., faces, landscapes). The general aim of such studies is to reveal visual qualities that humans find universally appealing (e.g., those related to color, form, or spatial arrangement) to demonstrate how common aesthetic preferences and associated emotional experiences are driven by predisposed neural structure. More simply, such fMRI studies investigate brain activity as it relates to participants' favorable responses to certain kinds of visual images, addressing the question of “What is *beauty* in the brain?”

**Figure 1:**
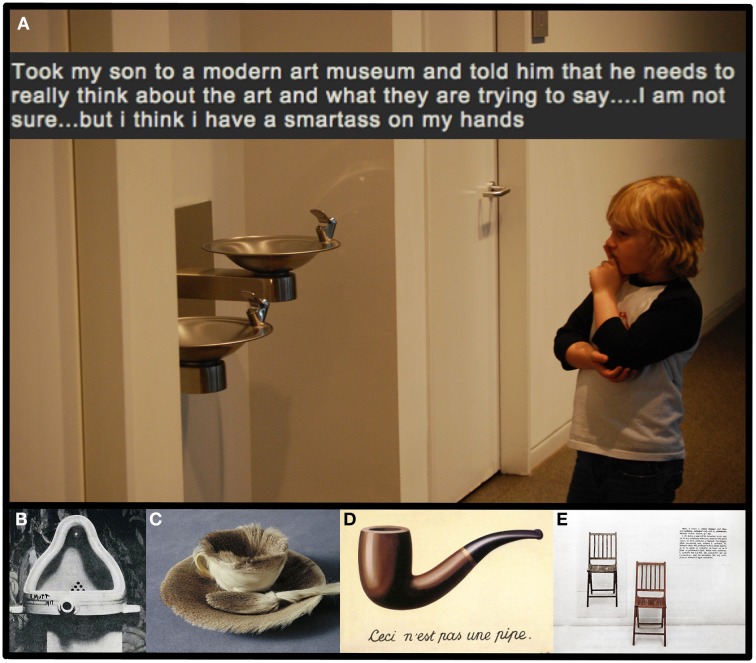
**The production and consumption of conceptual art tap into an interpretative stance that is older, and more basic than the genre itself. (A)** *Took my son to a modern art museum*, Simon1972 (2013). The moment captured in this photo and caption suggests that understanding the difference between art objects and utilitarian ones-along with the critical stance associated with conceptual art-can appear relatively early in childhood. **(B–E)** Uses of both reduction and contrast can be seen in iconic conceptual art pieces from a variety of time periods and movements (e.g., Dada, Surrealism, Minimalism, and contemporary Conceptual art). Works like **(B)** *Fountain* by Duchamp (1917) and **(C)** *Object* by Oppenheim (1936) place simple objects in novel contexts. Whereas **(D)** *The Treachery of Images* by Magritte (1928–1929) and **(E)** *One and Three Chairs* by Kosuth (1965) assemble representations of common objects in distinct formats. The use of reduction and contrast lend themselves to meaningful visual images that are salient and psychologically digestible. (All low-resolution images of art works were obtained from Wikipedia with reproduction here constituting fair use for academic and educational purposes in an open access journal.)

Although past research has provided valuable insight into the neural bases for aesthetic preference, research in neuroaesthetics need not be limited as such. To move beyond the beautiful, neuroaesthetics might more deeply consider conceptual art. Indeed, a distinguishing feature of conceptual art is that it seems not to be primarily concerned with beauty but instead with *meaning*. Defining conceptual art, Joseph Kosuth said, “Conceptual art, simply put, has as its central tenet an understanding that artists work with meaning, not with shapes, colors, or materials… The task for artists is to put into play works of art unfettered by the limited kinds of meanings which objects permit” (Kosuth, 1996, p. 407). How, in practice, do conceptual artists “work with meaning” though? Many iconic works of conceptual art employ two general devices in the service of working with meaning: *reduction* and *contrast*. Reduction refers to a process of simplification that frequently results in works that focus on individual things or, non-coincidentally, individual concepts. These can be concrete concepts like cups, pipes, and chairs, or abstract ones like art, space, and language. Contrast, refers to a method by which artists place an object within a specific context, or imbue it with specific features for the purpose of making semantic distinctions salient (See Figures 1B–E). For example, placing a urinal in a gallery, hair on a cup, or a physical chair beside its photograph, respectively underlines semantic distinctions between *utilitarian objects* and *art objects*, *living things* and *nonliving things*, and *the real* and the *represented*.

Similar devices are used in neuroscience when we attempt to operationalize and investigate some aspect of cognition. Reduction needs no introduction for the scientist. Any scientific problem is made simpler; a system is studied with respect to its parts. Like the conceptual artist, a cognitive neuroscientist interested in meaning starts with basic concepts or objects. Cognitive neuroscience work is contrastive or relational in nature as well. In a typical fMRI study, meaningful contrasts are planned such that any resulting neural activation that differs significantly between experimental conditions can be interpreted. For example, the neural response to reading nouns may be compared to that for verbs, or recognizing tools may be compared to recognizing animals. Reductionist and contrastive methods can also be applied to basic abstract concepts like space, time, and causality (Kranjec et al., 2012). This general approach, that defines concepts in terms of relations to other concepts, suggests that at its core, cognitive neuroscience aims to show how the brain makes meaning of the world. Studies tell us about the neural architecture that instantiates domain-specific processing while honing our definition of that domain. Cognitive neuroscience routinely asks questions like “What is a face?… a word?… a place? … or an object?” The meaning of such concepts is determined by the interpretation of empirical data.

## What is art?

Beyond beauty, a more fundamental question neuroaesthetics might ask is: “What is *art* in the brain?” Understanding how the brain responds to art, or to the artistic, *as such* is an important question that has been largely ignored. An object or image need not be judged as “beautiful” to be appreciated as art (Conway and Rehding, 2013). Likewise, beautiful things are not always art (Zaidel, 2015).

Chatterjee (2013) writes, “to my knowledge there has not been any serious attempt to think about the science of conceptual art” (p. 147). However, rather than thinking as “some people might … that conceptual art distracts scientists from getting to the very essence of art” (Chatterjee, 2013, p. 149), I believe conceptual art has the potential to ground neuroaesthetics research that seeks to investigate the meaning of art. This can be accomplished by using an analogous program to the one used to define beauty from the perspective of the brain. It has been suggested that a kind of “parallelism” (Chatterjee, 2011) exists between cognitive neuroscience and art. Zeki (1999) described how, as experts in visual representation, artists discovered organizing principles of visual perception long before empirical scientists. Similarly, conceptual artists have already asked many of the questions that cognitive neuroscientists should be asking about art and the mental structure of concepts more generally (Kranjec, 2013). Minissale (2012) describes four qualities of conceptual art that could guide research by cognitive neuroscientists. These include the (1) “puzzling” nature of conceptual art, (2) the interplay of “visible and invisible” aspects of conceptual art, (3) “intertextuality” or the manner in which conceptual art frequently references other works, and (4) the “conceptual complexity” of conceptual art. With respect to these four qualities, a complicated work of conceptual art might engage various neurocognitive systems related to problem solving, memory, and categorization. Presumably, most personal encounters with particular works of conceptual art would have a sledgehammer effect on neural systems that participate in semantics. Context, emotion, and individual knowledge or preference would all be expected to modulate any such neural response.

Perhaps neuroaesthetics has mostly ignored conceptual art because experimental approaches are generally not well suited to unpacking the complex layers of intent and reference that define many individual works. In general, methods in cognitive neuroscience are limited. This is why pioneering cognitive neuroscientists interested in metaphor comprehension did not design their preliminary fMRI studies using the full text of *Moby Dick*. Similarly, a burgeoning neuroaesthetics of conceptual art might choose to avoid a complex stimulus like Joseph Beuys' *I Like America and America Likes Me* (1974) where, among other things, the artist shares a room with a coyote for 3 days. Yet, despite limitations in neuroscience methods, it may be possible for meaning in art to be investigated reductively while still using conceptual art as a model. And as metaphorical thinking is used beyond its deployment in literature and poetry (Lakoff and Johnson, 2008), the production and consumption of conceptual art tap into cognitive processes more basic than the genre itself (see Figure 1A). To create a neuroscience of conceptual art we need to set the agenda much as the first conceptual artists did. Empirical approaches will need to (1) focus on less complex, object-oriented conceptual art (2) broaden the scope of objects that tend to be included in aesthetic research (including non-art objects), and (3) conduct research probing general processes (e.g., reduction and contrast) and ideas (e.g., “What is art?”) associated with conceptual art rather than their specific products. Artists like Marcel Duchamp and Andy Warhol were able to transform mundane objects into art by injecting them with artistic intent. Minimalists like Sol Lewitt and Mel Bochner refined conceptual questions relating to process, objecthood, and representation. These artists provide a good point of entry for inspiring empirical investigations of conceptual art.

What does it take for an object to be perceived as art in the mind of a human being beyond formal attributes like shape, pattern, or color? Is the distinction triggered by a particular stance an observer takes when responding to objects in a specific physical, functional, or social context, like a museum, kitchen, or religious ceremony? FMRI could explore the extent to which the difference between a special “art object” and an ordinary or “mundane object” is meaningful at the neural level. Specifically, one could investigate whether the neural bases for conceptualizing artistic intent, and perceiving artistic features, can be dissociated during object processing. In the cognitive neurosciences much is known about “normal” object processing in terms of naming, form, and function. Yet how object processing may differ when participants search for artistic intent and meaning is unknown.

Research would not require familiarity with conceptual art or be limited to the context of the modern and contemporary high art world. The production of art has spanned human history and proliferated across the globe. Throughout this process art objects have been created alongside more common, utilitarian objects. What defines the difference between “art” and mundane material objects? Perhaps shaping an art object involves taking something out of its everyday context and somehow making it special (Dissanayake, 1992). According to Dissanayake, art traditionally “makes special” those objects that are critical for survival. For example, objects like utensils and clothing are enhanced by art across all cultures. Such enhancement has the adaptive function of acknowledging the realities of survival, while making them less mundane. A neuroaesthetics of conceptual art could aim first at revealing the brain structures associated with processing what is special about such objects, beyond beauty. In this manner we may better understand how an object becomes construed and perceived as art by the observer and begin to understand the neural processes at work when identifying, perceiving, and analyzing an object as a work of art. Current cognitive neuroscience methods can play a part in delineating the meaningful conceptual boundaries between art and non-art while keeping other traditional aesthetic variables constant.

It may be more productive to investigate the processes not the products of conceptual art. Rather than attempting to map the vast neural network involved in interpreting specific, semantically complex works of conceptual art, neuroscientists might begin to think more like some conceptual artists. This way we can design experiments that allow participants to think conceptually about art as well, but in a relatively constrained manner. This can be done by reducing conceptual and phenomenological complexity: what many scientists and conceptual artists do best.

### Conflict of interest statement

The author declares that the research was conducted in the absence of any commercial or financial relationships that could be construed as a potential conflict of interest.

## Footnotes

^1^Although, *Neural correlates of interspecies perspective taking in the post-mortem Atlantic Salmon: An argument for multiple comparisons correction* by Bennett et al. (2009) is arguably the most successful work of conceptual art by neuroscientists.
